# Biomarkers as Predictive Factors of Anti-VEGF Response

**DOI:** 10.3390/biomedicines10051003

**Published:** 2022-04-26

**Authors:** Miriam Bobadilla, Ana Pariente, Ana I. Oca, Rafael Peláez, Álvaro Pérez-Sala, Ignacio M. Larráyoz

**Affiliations:** 1 Biomarkers and Molecular Signaling Group, Neurodegenerative Diseases Area Center for Biomedical Research of La Rioja, CIBIR, 26006 Logroño, Spain; mbobadilla@riojasalud.es (M.B.); apariente@riojasalud.es (A.P.); aioca@riojasalud.es (A.I.O.); rpelaez@riojasalud.es (R.P.); aperez@riojasalud.es (Á.P.-S.); 2 Department of Nursing, GRUPAC, University of La Rioja, Duquesa de la Victoria 88, 26006 Logroño, Spain

**Keywords:** age-related macular degeneration, ranibizumab, aflibercept, brolucizumab, anti-VEGF, SNPs, microRNAs, proteomic, metabolomic, antiangiogenic therapy

## Abstract

Age-related macular degeneration is the main cause of irreversible vision in developed countries, and intravitreal anti-vascular endothelial growth factor (anti-VEGF) injections are the current gold standard treatment today. Although anti-VEGF treatment results in important improvements in the course of this disease, there is a considerable number of patients not responding to the standardized protocols. The knowledge of how a patient will respond or how frequently retreatment might be required would be vital in planning treatment schedules, saving both resource utilization and financial costs, but today, there is not an ideal biomarker to use as a predictive response to ranibizumab therapy. Whole blood and blood mononuclear cells are the samples most studied; however, few reports are available on other important biofluid samples for studying this disease, such as aqueous humor. Moreover, the great majority of studies carried out to date were focused on the search for SNPs in genes related to AMD risk factors, but miRNAs, proteomic and metabolomics studies have rarely been conducted in anti-VEGF-treated samples. Here, we propose that genomic, proteomic and/or metabolomic markers could be used not alone but in combination with other methods, such as specific clinic characteristics, to identify patients with a poor response to anti-VEGF treatment to establish patient-specific treatment plans.

## 1. Introduction

Age-related macular degeneration (AMD) is the main cause of irreversible vision loss worldwide and accounts for 8.7% of all blindness worldwide [1,2], being the most common in developed countries, particularly in people older than 60 years [3,4,5]. Its prevalence is likely to increase as a consequence of exponential population aging reaching 288 million by 2040 [6]. The current gold standard treatment for neovascular AMD is intravitreal anti-VEGF injections. The majority of patients, ≈80–90%, maintain vision with anti-VEGF treatment, with approximately 30% of these showing a significant improvement. Nevertheless, 40–50% do not show any treatment benefits and continue losing vision [7]. There are multiple reports describing the differential response rates to anti-VEGF therapy for neovascular AMD [3,8,9,10,11]. The ability to predict how a patient might respond to treatment and how frequently retreatment might be required would enable clinicians to plan treatments schedules more appropriately, mitigating unwanted side effects and financial costs by reducing unnecessary monitoring visits [3]. In this review, we discuss the great controversy between genetic, proteomic and metabolomic studies carried out in AMD ranibizumab-treated patients, and we propose that genomic, proteomic and/or metabolomic markers could be used not alone but in combination with other methods, such as specific clinic characteristics, to identify patients with a poor response to anti-VEGF treatment to establish patient-specific treatment plans.

## 2. Macular Degeneration

**Figure 1:**
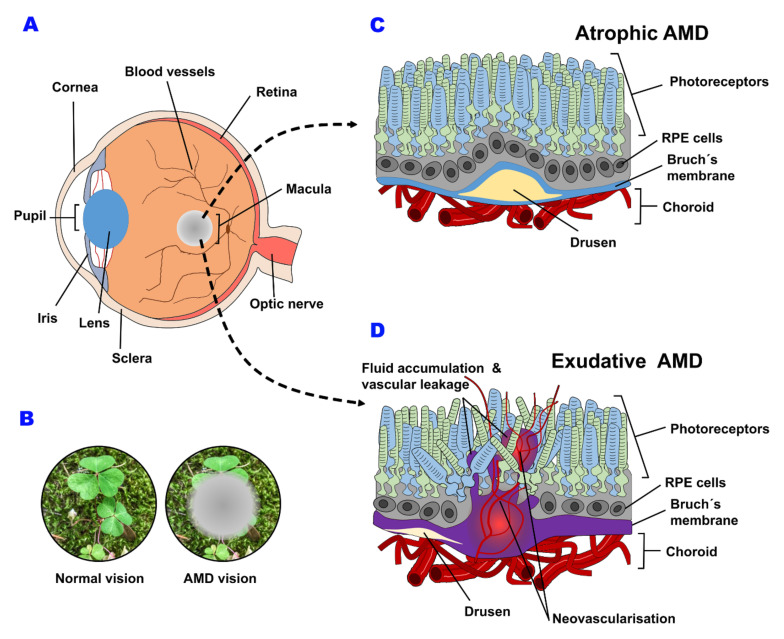
Schematic representation of an AMD eye (**A**) and the functional effects on vision capability (**B**) and structural retinal abnormalities associated with each subtype of AMD disease (**C**,**D**). Drusen accumulation, Bruch´s membrane alteration and RPE modifications are classical retinal changes associated with atrophic AMD (**C**), while neovascularization, fluid accumulation and vascular leakage are typical markers of exudative AMD (**D**).

AMD affects the macula (Figure 1A), and individuals lose central vision in one or both eyes (Figure 1B). At the onset, the disease is characterized by the presence of drusen (Figure 1C,D), which may progressively accumulate and predispose affected persons with others toward the advanced forms of the disease [12,13,14]. Severe vision loss occurs in the late stages of the disease, divided into neovascular, exudative or “wet” AMD and atrophic or “dry” AMD, both stage IV diseases according to the Age-Related Eye Disease Study classification (Figure 1C,D) [9]. The dry form is characterized by localized regions of atrophy of the retinal pigment epithelium (RPE) with areas of photoreceptor cell death. Conversely, neovascular AMD involves the growth of new blood vessels from the choroid capillary across the Brunch’s membrane/RPE into the neural retina. These newly formed blood vessels show increased permeability, which can lead to the accumulation of serous fluid or blood under the RPE or between the RPE and the sensory retina [15,16]. This process is accompanied by inflammation, hemorrhage and progressive fibrosis with a resultant significant loss of vision [17].

Although geographic atrophy accounts for the majority (90%) of cases of advanced disease, neovascular AMD causes more cases of legal blindness, because it is usually rapidly progressive, resulting in a loss of acuity and distortion of shape. Furthermore, approximately 10–20% of patients with dry AMD eventually progress to the exudative form, which is responsible for most of the estimated 1.75 million cases of advanced AMD in the US [7,15].

## 3. Incidence, Prevalence and Risk Factors

Race is an important factor in both the incidence and the prevalence of AMD [18]. Thus, several studies have reported that the incidence rate is higher in Caucasians compared to African Americans [15]. Furthermore, it has also been described that incidences in Asians are between the above two rates, although it seems that incidences are increasing in this population. The prevalence of AMD in Caucasians has been found to be almost twice as high as that in Asians. Caucasians have a higher prevalence of both early and late AMD compared with Africans, and dry AMD has been described to be more prevalent in Caucasians than in Asians and Africans [19]. Finally, taking into account the sex, data from several large population-based studies also have suggested that women are at increased risk for wet AMD compared to men [15,20].

Such as occurs in other chronic age-related diseases, AMD is a multifactorial disease with associated age, environmental, systemic and genetic factors [13,21]. Age is the major risk factor for AMD. The prevalence of all forms of AMD increases significantly with age. It affects ~17% of all individuals between the ages of 55 and 64, and the prevalence rises to 37% in those 75 or older in the United States [22]. Tobacco smoking is also a consistently identified environmental risk factor [23,24,25], as well as hypertension [26], cardiovascular disease [27] or high body mass index [28], among other factors [29].

Different studies have reported that dyslipidemias and metabolic dysfunction are associated with AMD [30]. The drusen deposited in AMD resemble the atherosclerotic deposits in vascular walls that are typically seen in high-risk cardiovascular patients, but studies on a possible association between AMD and atherosclerosis have yielded conflicting results to date [31,32]. Multiple genetic risk alleles for the susceptibility and progression of AMD have been identified in recent years (Table 1) [19]. The two most important ones are polymorphisms in *CFH* (complement factor H) and *ARMS2* (age-related maculopathy susceptibility 2) [33,34,35]. These two alleles together account for up to 45% of the risk of developing AMD [36,37]. Subsequently, single-nucleotide polymorphisms (SNPs) in other complement components have also been associated with AMD: complement factor 2, B (CFB) [38,39], 3 (C3) [40,41] and I (CFI) [42]. Associations between other genes and AMD development have been suggested (Table 1).

## 4. Treatment Options for AMD

Nowadays, the best treatment for AMD depends on several factors, including the stage of the disease. Either way, in all disease stages, the elimination of risk factors—for instance, smoking cessation—is especially appropriate [59]. Dietary supplements for AMD have been widely discussed in the literature [60]. However, the supplementation was found to have only a small effect on the intermediate stage, and no effect was found in the early or late stages of the disease [61]. The early detection of AMD may thus help motivate the patient to change lifestyle habits that promote the progression of the disease.

At present, no effective treatment options are available for the dry late form of AMD. All of the clinical trials carried out to date have not yielded positive results, including recent ones that have focused on modulators of the complement system [37]. However, there are different treatment options for the exudative late form of AMD, and therapy for this stage has undergone a rapid evolution over the past several years [36,62,63].

The precise cause of neovascular AMD is uncertain. Normally, the endothelial cells that line the ocular blood vessels are resistant to neovascular stimuli, and this quiescent state is attributed to a balance between proangiogenic (VEGF) and antiangiogenic factors. It is known that, in the development of the neovascular form of AMD, there is a balance between angiogenic and antiangiogenic factors, and the loss of this balance favors the development of blood vessels de novo [64]. This process involves endothelial cell migration, proliferation and survival, as well as vascular maturation, vessel wall remodeling and degradation of the extracellular matrix [62]. The unbalance can be produced for various reasons. Drusen deposits between Bruch’s membrane and the RPE may contain bioactive fragments of complement components (C3a and C5a), and these substances induce a VEGF expression with significant chemotactic activity that further invites inflammatory cells to the macular region [63]. Similarly, inflammation may be associated with angiogenesis, because inflammatory cells produce cytokines such as interleukin-1, which also induce VEGF [62]. Finally, this VEGF upregulation may account for the development of the new blood vessels [65,66], and for this reason, it has become the main target of some molecules in neovascular AMD therapy.

At present, there are few intravitreal injections of the VEGF antagonist available and used for the treatment specifically of neovascular AMD, such as Pegaptanib sodium (Macugen^®^), Bevacizumab (Avastin^®^), Ranibizumab (Lucentis^®^), Aflibercept (Eylea^®^), Brolucizumab-Dbll (Beovu^®^) and Faricimab-Svoa (Vabysmo^®^). The five approved drugs are injected directly in the vitreous body of the eye by intravitreal administration. Multiple other biosimilar drugs are likely to become available in the next few years [37,67].

Pegaptanib sodium: Pegaptanib is an aptamer that was approved in 2004 and was the first AMD therapy to selectively block VEGF [68]. Pegaptanib was well-tolerated but failed to offer a distinct advantage in the visual outcome, possibly because it selectively binds only to VEGF165 [68]. It has largely become obsolete since its approval in 2004, as more effective anti-VEGF treatments have been introduced [69].

**Figure 2:**
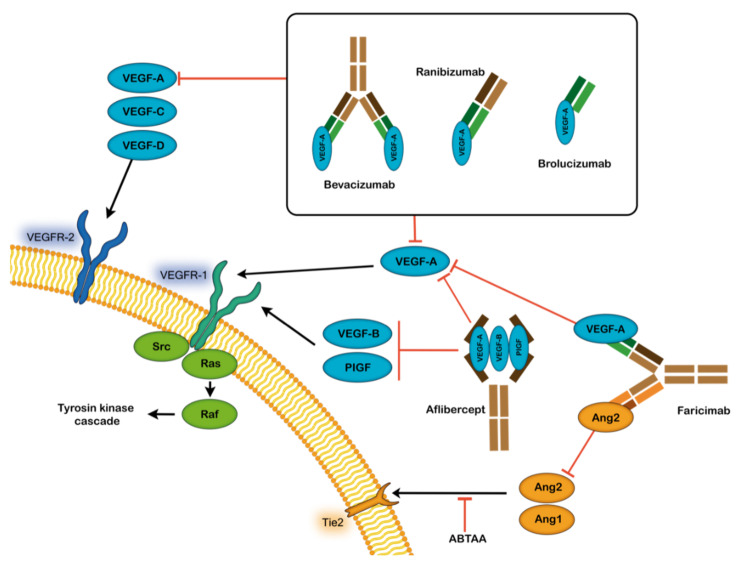
Summary of the currently available anti-VEGF treatments for neovascular age-related macular degeneration: bevacizumab (off-label), ranibizumab, brolucizumab, faricimab and aflibercept. VEGF-A, B, C and D: Vascular endothelial growth factor A, B, C and D; VEGFR-1 and 2: Vascular endothelial growth factor receptors 1 and 2; PlGF: Placental growth factor; Ang 1 and 2: Angiopoietin 1 and 2.

Bevacizumab: Bevacizumab is a full-length monoclonal antibody (149 kDa) [70] (Figure 2). Initially approved in 2005 for treating colon cancer, it has been used off-label since 2005 as a treatment for neovascular AMD. This drug is a full-length, humanized, recombinant monoclonal antibody against all isoforms of vascular endothelial growth factor A (VEGFA) [66,71]. It is significantly less expensive than many other anti-VEGF treatment options, as each vial can be fractionated into smaller doses for ocular use. Bevacizumab has been shown to be noninferior to ranibizumab in comparative studies and is often used as a first-line agent [66,72]. The durability and drying efficacy of bevacizumab have been shown to be less than other agents in select patients.

Ranibizumab: Ranibizumab is an anti-VEGF-A affinity-matured humanized monovalent monoclonal antibody fragment (48 kDa) that targets all isoforms of VEGFA (Figure 2) [73,74]. This drug was designed for ocular use [75], and its use was approved by the FDA (Food and Drug Administration) in 2006. Ranibizumab showed improvement in vision in the MARINA study and in photodynamic therapy in the ANCHOR study on classic lesions [71]. Despite its promising clinical study results, real-world studies have been challenged to replicate the vision gain those patients experienced in the clinical studies of ranibizumab over the long term.

Aflibercept: Aflibercept, also named VEGF Trap-eye, is the most recent member of the anti-VEGF family approved in 2012 by the FDA. It is a recombinant fusion protein (115 kDa) comprising the second Ig domain of human vascular endothelial growth factor receptor 1 (VEGFR1), the third Ig domain of human VEGFR2 and the Fc region of a human IgG1 (Figure 2) [71,76]. This drug has been recently developed to offer a more potent and prolonged anti-VEGF effect and was approved by the FDA in November 2011 [77].

Aflibercept is designed to target VEGFA with a higher affinity than Bevacizumab and Ranibizumab, vascular endothelial growth factor B (VEGFB) and placental growth factor PlGF [78]. VEGF Trap-eye may be considered an attractive alternative to other anti-VEGF agents, because it has similar results to ranibizumab and bevacizumab with a longer duration of action. For the first time, an anti-VEGF drug can be given at 8-week intervals with results comparable to ranibizumab given every 4 weeks. Aflibercept was generally well-tolerated in the VIEW I and II studies, and the ocular adverse events were similar to those of ranibizumab or bevacizumab [77].

Brolucizumab: Brolucizumab, which was approved in 2020 by the FDA, is a single-chain antibody fragment that is comprised of the variable domain of the monoclonal antibody joined by a short flexible linker peptide that provides stability (26 kDa) [79] (Figure 2). The absence of the Fc region and the small molecular size of scFvs are advantageous from a pharmacokinetic, as well as a manufacturing, standpoint [80]. Brolucizumab was designed to target VEGFA and was tested in the OSPREY trial and in the HAWK and HARRIER pivotal trials. The results showed that brolucizumab was a highly effective therapeutic molecule with concentrated molar dosing that showed important gains in visual acuity, superior fluid resolution and longer effects than other anti-VEGF treatments [79].

Faricimab-Svoa: Faricimab is a new bispecific, anti-VEGFA and anti-angiopoietin 2, antibody derived from Roche’s CrossMab technology [81]. The TENAYA and LUCERNE studies evaluated the effects of faricimab and aflibercept in patients with DMA, demonstrating the noninferiority of faricimab compared with aflibercept. The key findings showed the greater durability of faricimab compared to previous anti-VEGF in clinical trials [82].

## 5. Predictive Factors of Anti-VEGF Resistance

The current gold standard treatment for neovascular AMD is intravitreal anti-VEGF injections every 1–3 months based on the clinical response [66]. Although anti-VEGF agents have shown important achievements in neovascular AMD treatment, some patients have a poor or nonresponse to the standardized treatment [83]. Resistance can occur at any time during the course of therapy, so anti-VEGF therapy may fail from the beginning or following an initial successful treatment period [84]. For this reason, studies focusing on the causes of anti-VEGF therapy resistance would be useful for developing novel strategies to improve the efficacy of antiangiogenic therapies.

An intravitreal dexamethasone implant is an alternative treatment for those AMD patients resistant to anti-VEGF therapies that have been used in other retinal pathologies, such as macular diabetic edema [85]. Dexamethasone, in combination with anti-VEGF treatment, showed better results in a small cohort of anti-VEGF-resistant patients, but larger studies should be done in order to validate dexamethasone intravitreal implants as an alternative therapy [86,87].

The majority of patients, ≈80–90%, maintain vision with anti-VEGF treatment, with approximately 30% of these showing a significant improvement. Nevertheless, 40–50% do not show any treatment benefits and continue losing vision [7]. Although the reasons are not clear, several factors such as age, choroidal neovascularization (CNV) lesion characteristics and several genes have been suggested as the main causes [9]. Furthermore, different authors have indicated that an important mechanism of resistance to anti-VEGF therapy is the activation of alternative PDGF-, FGF-related and other angiogenic pathways such as angiopoietin [88,89]. For this reason, some anti-VEGF molecules such as Aflibercept and Faricimab are bispecific against VEGF and PlGF and angiopoietin-2, respectively, although these molecules have not shown better results than those that only inhibit VEGF [82].

There have been multiple reports describing the differential response rates to anti-VEGF therapy for neovascular AMD [3,8,9,10,11]. The ability to predict how a patient might respond to a treatment and how frequently retreatment might be required would enable clinicians to plan treatments schedules more appropriately, mitigating unwanted side effects and financial costs by reducing unnecessary monitoring visits [3]. The research into the genetics of AMD patients may help to predict the outcome of a particular therapy and, thus, lead to personalized medicine.

Biomarkers can be useful in clinical practice for detecting disease, monitoring its progression, the evaluation of treatment efficacy and risk assessment. Biomarker testing is an important step toward personalized medicine in many diseases such as cancer but, also, in age-related macular degeneration (AMD) [90].

### 5.1. Predictive Ranibizumab Biomarkers Based on Clinical Characteristics of Patients

Although most AMD patients responded to anti-VEGF therapy, some of them have an insufficient response. Some authors describe non-responders with initial clinical characteristics that might be informative for managing their treatment [91] and, if necessary, choose other anti-VEGF drugs or photodynamic therapy (PDT) at an earlier time point, which may improve the prognosis and avoid inefficient treatment.

The relevance of different optical coherence tomography (OCT)-derived parameters as prognostic and retreatment criteria during the treatment of AMD is controversial, and some authors have shown that the accurate measurements of retinal thickness may fail to correlate with visual function or to predict visual outcomes [92]. Furthermore, Suzuki et al. compared the initial characteristics of 141 responder and non-responder patients. The non-responders were identified 12 months after the first intravitreal ranibizumab (IVR) either by best-corrected visual acuity (BCVA) or by the fundus findings, including OCT images. The authors found that nine eyes (6.4%) were non-responders, as judged by both BCVA and the fundus findings, and fibrovascular pigment epithelial detachment was the only predictor for a nonresponse [91]. Currently, clinical research has focused on the identification of clinically relevant OCT-based parameters in retinal morphology that allow identify responder or non-responder patients [93].

Another studied characteristic that could be important to distinguish responder from non-responder patients is the leukocyte telomere length [94]. Telomeres located at the ends of chromosomes are involved in genomic stability and play a key role in various age-related diseases. Weng and collaborates found, in a study with 197 AMD patients and 259 healthy controls, that the leukocyte telomere length played an important role in the pathological mechanisms of AMD [95]. In accordance with these results, Fursova et al. analyzed the responses of 110 AMD patients to anti-VEGF therapy, depending on the functional and anatomical parameters of the retina and leukocyte telomere length. An association of the leukocyte telomere length with the response to treatment was found in AMD patients, but further studies on a larger patient cohort would be necessary to validate them [96].

Recently, Diack and colleagues described that the response to ranibizumab treatment and progression of the disease were functions of several clinical characteristics. AMD patients of the ANCHOR (426), MARINA (716 patients) and PIER (180 patients) clinical trials were included in this study [97]. The authors created a model-based meta-marker with a capacity for predicting the response to ranibizumab for a group of patients based on seven baseline characteristics: visual acuity, age, leakage size, central retinal lesion thickness, presence or absence of a cyst, type of CNV and size of the pigment epithelium detachment. A composite score of these seven baseline characteristics was used to categorize the response to ranibizumab treatment, obtaining an AUC of 0.86, although the model was able to partially explain the results [97].

### 5.2. Genomics, Proteomic and Metabolomic Predictive Ranibizumab Biomarkers

The current work on genetic and molecular biomarkers for treatment response in AMD is still exploratory, and precision medicine for AMD is not yet ready for implementation in clinics [98]. The great majority of studies are focused on the search for SNPs, analyzing the genes associated with the risk factors of AMD. Today, there is no ideal biomarker to serve as a predictive response to ranibizumab therapy, and there is great controversy between the findings of different authors. This fact could be explained, perhaps, by the great variability of the patients included in the studies (duration of treatment, number of administered doses, samples, nationality, etc.). However, some described results look promising, and further studies on a larger patient cohort would be necessary to validate them (Table 2).

#### 5.2.1. Predictive Ranibizumab Biomarkers in Saliva

DNA extraction from saliva samples has been used in several AMD pharmacogenetic studies due to the easiness and the low invasiveness of sample collection [99,100,101,102,103,104,105,106,107].

- Complement System

The most studied polymorphisms in mouthwash samples are *CFH* SNPs [99,100,101,102,103,104,105]. However, the implication of this gene in the anti-VEGF therapy of exudative AMD patients is controversial. Some authors found no significant correlation between *CFH* rs1061170 (Y402H) polymorphism and the treatment response [101,104]. By contrast, Brantley et al. described in a study of 86 patients an association between visual acuity (VA) and this SNP. VA improvement was shown in approximately 50% of the patients with the non-risk genotype of *CFH* rs1061170 TT (5/10 patients) and the TC genotype (31/57 patients) after an intravitreal bevacizumab treatment [99]. Only 10.5% of the patients carrying the risk genotype CC had visual outcomes (2/19 patients), and this result is in accordance with that described in a study of 94 patients treated with ranibizumab [99,107]. Nevertheless, there are also inconsistencies among the studies describing a relationship between *CFH* Y402H and the anti-VEGF treatment response in AMD patients. A more recent study suggested that patients with the risk genotype CC presented a better anatomical response after ranibizumab compared to those with the TT genotype in a study composed of 403 patients, but the significance disappeared after Bonferroni correction [103]. Similarly, in 69 AMD patients treated with PDT, patients with the non-risk genotype presented worse VA outcomes when compared with those carrying the risk allele (TC or CC) [100]. Moreover, Lee et al. found no relation between this SNP and VA improvement in a study of 156 patients but pointed out that patients carrying the risk genotype CC were 37% more likely to need another ranibizumab injection than the TT genotype after the ninth month [105].

Differences in the treatment regimen used could explain the different relations of the *CFH* Y402 genotypes and anti-VEGF response. It has also been suggested that ethnicity may appear to be important in the association or not of *CFH* rs1061170 with the anti-VEGF treatment response. Its involvement has been reported in Caucasian populations [101,102,104,105,107,108], whereas no effect has been described in Asian populations [101].

Other polymorphisms of *CFH* associated with the anti-VEGF response less frequently tested in Caucasian populations are rs1048663, rs3766405, rs412852, rs11582939 and rs1066420, with worse visual outcomes carrying the risk allele in samples of 68 patients [102]. Moreover, patients with the protective genotype of rs800292 (AA) had a better basal VA, but the outcome after IVR was higher in those with the risk allele (GG and GA) in a cohort of 403 patients [103]. *C3* rs2230199 [102,107] and *CFB* rs12614 [103] polymorphisms have been also tested in mouthwash samples of AMD patients, and no significant relationship has been found between these SNPs and the anti-VEGF response, although patients with the CT genotype of *CFB* rs12614 had a tendency to improve the VA after treatment [102,103].

- ARMS2

The implication of *ARMS2* rs10490924 (A69S) in the response to anti-VEGF treatment has been also widely tested in saliva samples [99,101,102,103,104]. Kitchens et al. found a significant correlations of the risk genotype rs10490924 TT with no response to the treatment in a cohort of 100 patients (9/16 patients) [104]. Brantley et al. also described a trend in patients with the risk genotype of a worse treatment response (6/15 patients) than patients with the TG (15/38 patients) and GG (17/33 patients) genotypes, but these differences were not significant [99]. Nevertheless, these results are not supported by other reports, where no correlation between this SNP and the anti-VEGF or PDT treatment response in exudative AMD patients has been found [100,101,102,103,107].

- *VEGFA* and related genes

Due to the importance of VEGF-A in the development of exudative AMD, several *VEGFA* SNPs have been also evaluated in mouthwash samples [103,105,106]. Only rs833069 has been described to be significantly involved in the ranibizumab treatment response in a study of 102 patients. In that study, anatomical improvements at 3 and 6 months after treatment and measured with the central subfield macular thickness were shown but without significant visual outcomes [101]. A trend has been observed among the variants rs699947 CC, rs833061 TT and rs1570360 GG and a higher probability of not responding to treatment, although these results are not significant [104]. No relationship has been found between rs833060, rs36208049, rs23648, rs59260042, rs2010963 or rs3025000 and the response to anti-VEGF treatment in AMD patients [103,104].

With respect to other *VEGFA*-related genes, Cobos et al. described an association of *VEGFR1* rs7993418 TC and TT variants with a better anatomical response after 12 months of treatment measured in terms of the central foveal thickness [103,104]. By contrast, *VEGFR2/KDR* (kinase insert domain receptor) SNP rs2071559 was not related to the ranibizumab response [101].

- SERPINF1

*SERPINF1* (serpin family F member 1) codifies a protein secreted by RPE cells that has antiangiogenic activity and inhibits endothelial cells migration [103,109]. Two SNPs of this gene, rs12603486 and rs1136287, have been correlated with the ranibizumab response in 403 exudative AMD patients. Patients carrying the A allele of rs12603486 were more likely to have a poor response. With respect to rs1136287, the CT and CC genotypes presented less anatomical responses measured in terms of the central foveal thickness [104].

- Other genes

Variability at the mtDNA A4617G site was studied by Chaudhary et al. in 70 treated AMD patients, but no correlation between the treatment and patient response was found [102]. Furthermore, *HTRA1* (HtrA serine peptidase 1) rs11200638 was investigated in a cohort of 102 neovascular AMD patients, but it was not correlated with the ranibizumab treatment response [101].

#### 5.2.2. Predictive Ranibizumab Biomarkers in Whole Blood

Human blood is the most vital and widely used specimen for human disease assessments, mainly due to its low invasiveness of the sample collection and its correlation with a variety of diseases. Numerous studies based on SNPs have been conducted in whole blood samples over the last few years, all of them focused on studying only genes related to the development of AMD [9,33,108,110,111].

- Complement Factor H

The implication of CFH in anti-VEGF therapy is controversial. Some authors found no significant correlation between *CFH* rs1061170 (Y402H) polymorphism and the treatment response [112,113,114,115,116,117]. Nevertheless, other authors observed that Y402H polymorphism from the CFH gene was associated with an increased likelihood of a functional response to treatment, while CC polymorphism was related to a poor response to ranibizumab both in Hispanic (49/67 patients) and Brazilian (3/38 patients) populations, considering a bad response as a decrease in the VA of five letters or more compared to the initial examination [33,118,119,120]. Other polymorphisms of *CFH* associated with the anti-VEGF response studied in whole blood samples are rs800292, rs1329428 and rs1410996. All of them are associated with poor anti-VEGF therapy response in AMD patients [33].

- ARMS2

The implication of ARMS2 in the response to the anti-VEGF therapy of AMD patients has been studied in the total blood [102,104,121]. As is the case with other genes, some authors found no relation between ARMS2 (A69A) SNP rs10490924 polymorphism and the ranibizumab response [102,114,115,116,121,122,123]. However, Abedi and collaborators observed that the presence of rs10490924 was associated with a poorer visual outcome for ranibizumab treatment in neovascular AMD. The response to treatment was measured by a loss of five letters or more in VA, by evidence of the persistence or recurrence of intraretinal or subretinal fluid on OCT or by the presence of new or persistent hemorrhage during the fundus examination [33,124]. According to the same criteria, Mohamad et al. described that, in a study on 96 AMD patients, A69A was also correlated with the response to IVR therapy based on the visual and anatomical outcomes in the Malaysian population (67/96 patients) [125].

Other polymorphisms of *ARMS2* associated with the anti-VEGF response are rs3750848 and rs1061170, but no correlation was found between these SNPs and the ranibizumab treatment response [116,121].

- *VEGFA* and related genes

Several *VEGFA* (rs3025039, rs699947 and rs833061) and *VEGFR*2 (rs2071559) SNPs have been also evaluated in whole blood samples [124,125,126]. In a prospective study involving 394 treatment patients, Parks et al. explained that the TT genotype for *VEGFA* rs3025039 was associated with a significantly higher chance of a visual gain of ≥15 letters at month 24 of the ranibizumab treatment in a Korean population [122]. Similar results were found for rs699947 SNP in a study with 94 patients; those expressing the AA genotype had a higher possibility of increasing their best-corrected VA [122,123]. Similarly, Lazzeri and colleagues observed that a ranibizumab treatment was significantly more effective in patients harboring the *VEGF-A*-2578GG allele, whereas patients carrying the *VEGF-A*-2578AA genotype revealed the absence of an early functional response to ranibizumab in a cohort of 64 patients treated for neovascular AMD with ranibizumab monotherapy [127]. Nevertheless, no correlation was found between the *VEGFR* rs2071559 polymorphism and treatment response [123].

- HTRA1

The implication of *HTRA* in the anti-VEGF therapy of exudative AMD patients is also controversial.

An association between rs11200638 and a poorer visual outcome after a ranibizumab treatment in neovascular AMD was described. The authors considered a poor response as a loss of five letters or more in VA compared to the initial examination [20,125]. Furthermore, Mohamad et al. also studied rs11200638 in a cohort of 145 Malaysian AMD subjects. Similarly, the mRNA levels in the *HTRA1* variant between the responder and non-responder groups were significantly different for the homozygous non-risk GG genotype, suggesting that it could contribute to the non-responders’ reactions to ranibizumab [125]. Furthermore, some authors found no correlation between the *HTRA1* promoter SNP rs11200638 polymorphism and ranibizumab response [118,124,125].

- OR52B4

Olfactory receptor 52B4 is a protein encoded by the *OR52B4* gene. Three different SNPS have been studied in this gene (rs4910623, rs323085 and rs1015893). Riaz et al. performed a GWAS analysis on 285 anti-VEGF-treated AMD patients, concluding that *OR52B4* rs4910623 SNP was associated with a poor VA response after 3 and 6 months of ranibizumab treatment [128]. Likewise, Wang and collaborators recently described rs323085 in the OR52B4 gene being associated with good anti-VEGF therapy responses, whereas rs4910623 and rs10158937 in the *OR52B4* gene were associated with poor anti-VEGF therapy responses in AMD patients. The response to treatment was measured by a loss of five letters or more in VA, by evidence of the persistence or recurrence of intraretinal or subretinal fluid on OCT or by the presence of new or persistent hemorrhage during the fundus examination [33].

- ApoE

The apolipoprotein E (*ApoE*) protein is commonly found in drusen, which is the earliest clinical hallmark of AMD. Altered expression levels of *ApoE* can contribute to the formation of drusen and the pathogenesis of AMD [33]. Tikellis and colleagues demonstrated a higher likelihood of the development of AMD in individuals with the *ApoE* ε2 allele polymorphism than with the ε3 and ε4 variants [129]. In the same way, different studies with 109 and 192 patients treated with IVR injections also demonstrated that the presence of the *ApoE* ε4 allele (9/38 patients) conferred significantly better visual outcomes, an improvement of five ETDRS letters, after anti-VEGF treatment than the ε2 allele (4/15 patients) in patients with neovascular AMD [126,130].

- Other genes

*KDR* rs52071559 and rs7671705, LDL receptor protein 5 (*LPR5)* rs3736228, frizzle class receptor 4 (*FZD4*) rs10898563, *PLG2G12A* rs2285714, hypoxia inducible factor 1 subunit α (*HIF1A*) rs11549465 and signal transducer and activator of transcription 3 (*STAT3*) rs744166 were studied by Smailhodzic and colleagues, but no correlation between the treatment and patient response was found in AMD patients [115].

#### 5.2.3. Predictive Ranibizumab Biomarkers in Plasma

Plasma is the largest component of the human blood in immunoglobulins, clotting factors, proteins albumin and fibrinogen, enzymes and water. The main function of plasma is the transportation of cellular nutrients and proteins to different parts of the body [131,132].

Only a few numbers of studies about the response of AMD patients to ranibizumab treatment have been conducted on plasma samples, and only SNPs were analyzed [133,134]. Yildiz and colleagues examined different SNPs in *CFH* (Y402H) and *VEGF* (rs2146323 and rs2146323) in a total of 109 exudative AMD patients and 70 controls. Although *CFH* Y402H SNP might be protective of AMD development in the Turkish population, none of the SNPs studied showed any effect on the ranibizumab response.

In another study, Wang and collaborates examined 12 SNPs within *CFH*, *HTRA1*, *IL-17*, *IL-23R*, cytochrome P450 family 3 subfamily A member 4 (*CYP3A*) and Leptin in 43 patients, 29 responders and 14 non-responders to an anti-VEGF treatment. A higher rate of G allele carriers was observed in the responders (4/29 patients) compared to the non-responders (0/14 patients) for the *IL-23R* SNP rs11465804. However, no differences were found in the rest of the analyzed SNPs [135].

#### 5.2.4. Blood Mononuclear Cells as a Source of Ranibizumab Predictive Biomarkers

Peripheral blood mononuclear cells are blood cells with round nuclei, such as monocytes, lymphocytes and macrophages [136]. Until last year, only SNPs had been analyzed in the vast majority of these studies [137,138,139,140,141,142]; nevertheless, recently, both microRNA and mRNA expression were described as possible predictive biomarkers of the response to anti-VEGF therapy [7].

- Complement System

Similar as described above, *CFH* SNPs have been extensively studied, and their utilization as predictive biomarkers of the response to anti-VEGF therapy is controversial. Some authors found no significant correlations between two *CFH* polymorphisms: rs1061170 (Y402H), rs800292 (I62V) and the treatment response [137,138,139,140,141]. However, Gourgoli and colleagues, in a cohort of 52 neovascular AMD patients, described that Y402H homozygous patients (CC; 40%) were less likely to respond to ranibizumab therapy compared to the heterozygous patients (TC; 80%). The treatment response was evaluated by comparing VA and OCT between the baseline and the end of the treatment [142].

*C3* rs2230199 polymorphisms have been also tested in blood mononuclear cell samples of AMD patients. Mouallem-Beziere et al. compared 300 neovascular AMD patients, with 68 resistant to ranibizumab therapy, that the GG genotype of *C3* SNP rs2230199 was significantly associated with the phenotype of large vascularized pigment epithelial detachment poorly responding to anti-vascular endothelial growth factor therapy [139].

- ARMS2

*ARMS2 rs10490924* SNP has been studied in blood mononuclear cells samples. Lezeviel et al. demonstrated in a cohort of 1216 AMD patients an association between the at-risk allele of *ARMS2/LOC387715* and a poor response measured by a loss of five letters or more in VA [140]. In the same way, Gourgouli and colleagues described in a total of 52 neovascular AMD patients that the *ARMS2*/A69S genotype, carriers of the risk variant, experienced significantly worse treatment outcomes compared to wild-type patients [142]. Nevertheless, some authors found no significant correlation between this polymorphism and the treatment response [138,139].

- Other genes

*ApoE* rs4420638, transforming growth factor β receptor 1 (*TGFBR1*) rs334353, *CF1* rs4698775, cholesteryl ester transfer protein (*CETP*) rs3764261 and *VEGFA* rs943080 and the response to ranibizumab treatment in AMD patients rs744166 were studied by Yamashiro and colleagues, but no correlation between the treatment and patient response was found in AMD patients [141].

- MicroRNAs

Different studies have already demonstrated that circulating miRNAs can be of use as markers for retinal diseases such as diabetic macular edema [143] and that their dysregulation is common in AMD, glaucoma and Alzheimer’s disease [144], although none of these studies have analyzed miRNAs in relation to the response to ranibizumab treatment. Oca et al. studied the transcriptomes from 59 wet AMD patients before undergoing ranibizumab treatment, and a set of miRNAs that were able to predict the ranibizumab response were identified [7]. Furthermore, numerous pathways regulated by this set of miRNAs were identified, including thyroid hormone signaling, transforming growth factor β (*TGF-β*) signaling, Hippo and adherent signaling, endocytosis and others. In addition, this miRNA set also regulates important AMD-related genes, such as *VEGF-A*, platelet-derived growth factor subunit A (*PDGFA*) and platelet-derived growth factor receptor α (PDGFRA), involved in the VEGF-signaling pathway, ECM–receptor interaction, cytokine–receptor interaction and the cell cycle [7]. Specifically, Oca and colleagues demonstrated that hsa-miR-20a-5p was differentially expressed between responder and non-responder patients in blood mononuclear cells samples. Poor responders were defined as patients showing less than a 25% reduction in OCT central retina thickness from the baseline, with persistent or new intraretinal fluid, subretinal fluid or less than five letters in visual acuity after VEGF therapy [7]. This finding could allow to predict, retrospectively, with good accuracy, a successful response to ranibizumab before the start of the treatment.

- mRNAs

A RNA-Seq differential expression analysis was performed on mRNAs extracted from peripheral blood mononuclear cells (PBMCs) of 59 wet AMD patients with good or poor responses to ranibizumab before treatment initiation [7]. Four mRNAs were found differentially expressed in good and poor ranibizumab responders. ENSG00000249572 (lnc-ADAMTS12-6) is a novel transcript affiliated with the long noncoding RNA (lncRNA) class located in chromosome 5 of the human genome [145]. ENSG00000161298 (*ZNF382*) belongs to the Krüppel-associated box (*KRAB*) domain zinc finger transcription factor (*KZNF*) family, which has been shown to regulate differentiation, proliferation and apoptosis processes and reduce the expression of matrix metallopeptidase 1 (*MMP1*) that is dysregulated in AMD [146,147]. ENSG00000226479 (*TMEM185B*) is a protein-coding gene belonging to the TMEM family. Lastly, ENSG00000198056 encodes a small subunit of the Primase protein (*PRIM1*). This primase regulates critical processes in the retina, and its function has been associated with the development of inherited retinal degeneration, such as recessive Retinitis Pigmentosa [148]. These results are interesting, because the candidate biomarkers are not related to the genes involved in the development of AMD, as described by most studies; however, studies on a larger patient cohort are necessary.

#### 5.2.5. Predictive Ranibizumab Biomarkers in Aqueous Humor

Aqueous humor (AH) is a clear fluid that stabilizes the ocular chambers, but it also provides nutrition, removes metabolic products and regulates eye homeostasis. Under pathological conditions, AH transports inflammatory cells and participates in drug distribution [149]. Few reports have been done in AH samples, perhaps because the collection of these samples is more invasive and complex. Proteomic and metabolomic studies have been conducted in AH samples, and they used to be associated with intravitreal treatment administration [150,151,152,153].

Inflammation is closely related to AMD evolution, so cytokines and chemokines represent interesting molecules to be evaluated in the eyes of AMD patients. Sakamoto et al. observed in a Japanese cohort of 48 AMD patients that many cytokines (C-X-C motif chemokine 1 (CXCL1), CXCL12, CXCL13, Monocyte chemoattractant protein 1 (MCP-1), C-C motif chemokine ligand 11 (CCL11), IL-6 and IL-10) were downregulated after two ranibizumab injections, while only MMP9 was increased after treatment [150]. These proteins remain elevated even one month after the last injection. All these cytokines, except IL-10, presented a correlation between the initial baseline cytokine concentrations before treatment and their levels before the third IVR [150]. These data are in concordance with other studies that have evaluated the cytokine levels after an alternative anti-VEGF treatment, bevacizumab [154]. However, another study with 19 AMD patients did not show cytokine or chemokine modifications after a ranibizumab treatment. Although many cytokines were altered in the AMD group, only CCL11 was upregulated after two IVR [151]. In addition, subsequent studies in a Japanese population showed that CXCL12 and IL-10 were able to predict the number of ranibizumab injections after 2 months of treatment [155]. Furthermore, Pongsachareonnont et al. found in a cohort of 30 AMD patients that poor ranibizumab responders presented high levels of placental growth factor (PLGF) and reduced levels of IL-7 in contrast to good responders [156,157].

**Table 2:** Summary of the predictive biomarkers.

Summary Predictive Biomarkers					
Sample	Biomarker Type	Symbol	Biomarker	Correlation Ranibizumab Response	Reference
Saliva	*SNPs*	*CFH*	rs1061170 CC	Negative	[99,100,102,107]
			rs1061170 TT/CT	Positive	
Saliva	*SNPs*	*VEGFA*	rs833069	Positive	[101]
Saliva	*SNPs*	*VEGFR1*	rs7993418	Positive	[103,104]
Saliva	*SNPs*	*SERPINF1*	rs512603486	Negative	[103]
			rs1136287	Negative	
Whole blood	*SNPs*	*CFH*	rs1061170 CC	Negative	[19,118,119,120]
			rs1061170 TT/CT	Positive	
			rs800292	Negative	
			rs1329428	Negative	
			rs1410996	Negative	
Whole blood	*SNPs*	*ARMS2*	rs10490924	Negative	[19,124]
Whole blood	*SNPs*	*VEGFA*	rs3025039 TT	Positive	[122]
Whole blood	*SNPs*	*OR52B4*	rs4910623	Negative	[128]
Whole blood	*SNPs*	*ApoE*	Ɛ4	Positive	[126,130]
Plasma	*SNPs*	*IL23R*	rs11465804	Positive	[134]
Blood mononuclear cells	*SNPs*	*CFH*	rs1061170 CC	Negative	[142]
			rs1061170 TT/CT	Positive	
Blood mononuclear cells	miRNA	has-miR-20a-5p	↑ has-miR-20a-5p	Positive	[7]
			↓ has-miR-20a-5p	Negative	
Blood mononuclear cells	mRNA	Lnc-ADAMTS12-6	↓ Lnc-ADAMTS12-6	Positive	[7]
			↑ Lnc-ADAMTS12-6	Negative	
Aqueous humor	Protein	*CXCL12*	CXCL12	Negative	[158]
Aqueous humor	Protein	*CCL11*	CCL11	Positive	[151]
Aqueous humor	Protein	*IL-7*	IL-7	Positive	[156,157]
Aqueous humor	Protein	*IL-10*	IL-10	Negative	[158]
Aqueous humor	Protein	*MCP1*	MCP1	Negative	[158]
Aqueous humor	Protein	*HGF*	HGF	Negative	[157]
Aqueous humor	Protein	*PLGF*	PLGF	Negative	[156,157]
Aqueous humor	Protein	*KRT8*	KRT8	Negative	[159]
Aqueous humor	Protein	*sVCAM*	sVCAM	Negative	[157]

↑ Increased levels. ↓ Decreased levels.

Several studies have shown an increased integrin expression in retinal neo-vessels [160], as well as the important role of integrins in cell–cell and cell–matrix interactions of the internal limiting membrane to keep the correct eye structure [161]. A pro-inflammatory environment in the eye is linked to AMD evolution. Inflammation promotes integrin expression and immune cell recruitment to tissues—for example, to the retina—in an integrin–ligand interaction-dependent manner (VLA4-VCAM1 and LFA1-ICAM1) [162]. It has also been described that integrins could mediate different growth factor-induced angiogenesis mechanisms [163], and therefore, different anti-integrin strategies are being tested currently in clinical trials against AMD [161].

Mantel and colleagues showed that Il-6 but also other proinflammatory and angiogenic molecules such as circulating vascular cell adhesion molecule 1 (sVCAM-1), IL-12p40, plasminogen activator inhibitor type I (*PAI-1*) and hepatocyte growth factor (*HGF*) were increased in incomplete responders to anti-VEGF therapy. The response was measured by the presence of intraretinal or subretinal fluid accumulation after at least 6 months of monthly intravitreal injections [157]. In accordance with other studies, CCL11 and MCP1 [155] but also other molecules (EGF and IP-10) presented altered levels in incomplete responder patients. As expected, members of the VEGF family of proteins presented reduced levels in both types of ranibizumab-treated patients [157].

Keratin 8 (KRT8) is a RPE keratin detected in AH exosomes of AMD patients [164]. This protein has been associated with activation of the reparative mechanism after stress oxidative injuries in RPE cells, although it also induces RPE cell death under prolonged stress in an epithelial–mesenchymal transition-dependent process [165]. Shin and colleagues found in 58 AMD and 46 control patients, classified according to the OCT findings, an increase in the KRT8 levels in the AMD patients group. Furthermore, after three IVR doses, the responders presented a significant decrease in the KRT8 level, whereas poor responders showed no level changes [159]. In summary, Shin and collaborators found an association between a decrease in the KRT8 levels and a better response to anti-VEGF treatment [159]. Recently, Kim et al. observed that the Angiopoietin-like 4 protein (ANGPTL4), a glycoprotein whose expression is associated with hypoxia [166], was upregulated in exudative AMD patients [167]. Furthermore, the ANGPTL4 baseline levels were correlated with the lesion area, visual acuity, CNV size and frequency of ranibizumab injections in the non-responder patients group [167].

One of the AH functions is to remove metabolic products [153]. For this reason, a metabolomic study was performed to evaluate the metabolic signatures in the AH of AMD patients [168]. Forty-five patients (20 control and 25 AMD) showed an increased glycolytic activity; mainly, tricarboxylic acid cycle metabolites such as citrate, isocitrate, succinate and α-ketoglutarate were significantly different in the AMD group. Glutamine and glutamate also showed differences between the control and AMD patients [168,169], as well as other metabolic pathways such as tyrosine, urea metabolites or lipids [170,171]. Curiously, not one of the evaluated metabolites was altered after IVR treatment, and only the central macular thickness was statistically significantly modified [168].

## 6. Limitations and Possible Future Directions

**Figure 3:**
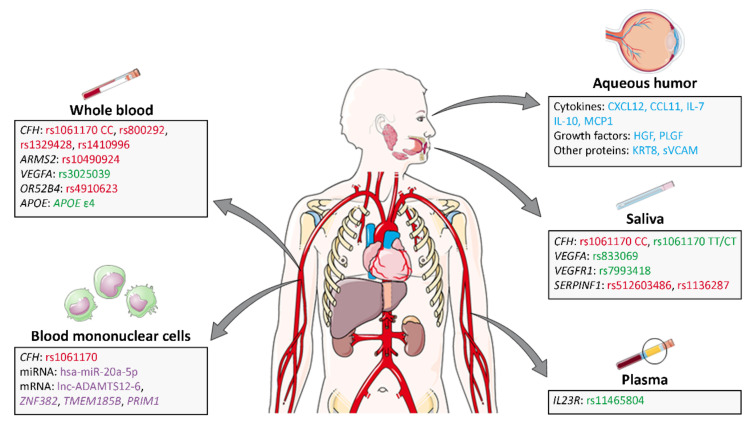
Summary of the main predictive biomarkers of the anti-ranibizumab treatment response described in whole blood, mononuclear blood cells, plasma, saliva and aqueous humor samples. In green, SNPs that contribute to a better anti-VEGF response; in red, SNPs associated with a worse response to anti-VEGF treatment; in blue, proteins whose levels differ between good and poor responders; in purple, mRNAs and miRNAs differently expressed in responder and non-responder patients. Images adapted from SMART Servier Medical Art (smart.servier.com, accessed on 27 March 2022).

Nowadays, an ideal biomarker to serve as a predictive response to ranibizumab has not yet been described. There is great controversy between reports, and this fact could be explained, perhaps, because there are no clear criteria to treat neovascular AMD and patients with variability in the duration of treatment, number of administered doses, samples and nationality are compared in the studies. In the same way, there are different criteria to define the response to the treatment. The majority of studies are focused on the search for SNPs, but few studies of miRNAs, DNA methylation, histone modification, proteomic and metabolomics have been carried out in AMD ranibizumab-treated samples (Figure 3). Unfortunately, the level of replication between the different reports is low, and the performances of the described biomarkers are still insufficient to warrant their implementation in the clinic. Recently, new methods based on the transcriptomic of blood cells and/or baseline characteristics of patients are showing promising results. We proposed that the combination of transcriptomic methods with genomic and proteomic methods would provide new opportunities to identify patients with a poor response to anti-VEGF treatment to establish better patient-specific treatment plans.

## Figures and Tables

**Table 1:** Relevant SNPs associated with AMD susceptibility and progression.

Genes Related to Susceptibility and Progression of AMD		
Gene	Single Nucleotide Polymorphism	Reference
*CFH*	rs1410996; rs1061170 (Y402H); rs800292 (V62I); rs2274700	[43,44,45]
*C2*	rs1042663; rs3020644; rs2072632; rs9332739; rs547154	[46,47]
*ARMS2*	rs10490924; rs3750848; rs10490923	[42,45,47,48]
*HTRA1*	rs11200638; rs932275	[48]
*CD36*	rs3173798; rs3211883; rs10499862; rs3173800; rs17154232	[49]
*APOE*	Ɛ2; Ɛ4	[50,51]
*PON1*	M55L; Q192R	[52]
*ERCC6*	C−6530 > G	[53]
*CFB*	rs641153; rs4151657; rs4151672; rs4151667	[46]
*C3*	rs1047286; rs3745565; rs171094; rs2230199 (R102G); rs11569536	[46]
*VEGFA*	rs699947; rs1413711; rs2010963	[54]
*CFI*	rs10033900	[42]
*VEGFR*	rs9319425; rs622227; rs2387632	[54,55]
*CXCR3CR1*	T280M; V249I	[56]
*TLR4*	D299G	[57]
*ELOVL4*	M299V	[58]

## Data Availability

Not applicable.
